# Doctor or Not Doctor?

**Published:** 1897-08-21

**Authors:** 


Vol. XXII.—No. 569.] [Aug. 21, 1897.

### Doctor or not Doctor?

A new medical session is before us, and "A Medical Man," among other correspondents, writing to the *Standard*, offers to intending doctors the advice which *Punch*, with the wisdom of a clearsighted modernity, offered a good many years ago to "those about to marry." "Don't," said Mr. Punch. "Don't be a doctor," says the medical man to young men, with pathetic earnestness. Choose any profession or calling you please, but whatever you do don't practise physic. Of course, it is open for the cynic to say that this is only one of the many weapons used, consciously or unconsciously, by all living creatures in the life-and-death struggle for survival. The medical man who writes to the *Standard* finds himself hard put to it to make a living, even as things are at present. He foresees that the struggle will become sterner and sterner with every succeeding year, if the present mad rush of students into the profession cannot by some means or other be checked.

The question is worth looking at, not perhaps so much from the standpoint of the struggling practitioner, for all of us must struggle in this world. Unhappy, indeed, is the lot of the man or the woman who is not called upon to struggle. There is nothing in all the world so good for any of us as a stiff hand-to-hand fight with destiny from the cradle to the grave. Nevertheless, when all this is admitted, it is still a very necessary thing indeed to exercise a careful foresight in the choosing of a calling.

Let it be granted, at the outset, that medicine is an excellent calling—for some people. It is honest, it is earnest, it calls forth most that is best in a man, and but little that is worst. The earlier part of it, the student life, is in many respects the most delightful of all periods to the young man who loves at once work, and culture, and progress. Perhaps the idea of "culture" needs here a little justification. But certainly, as Huxley long ago pointed out, and as thousands of our most highly cultivated men now admit, for stern and solid mental culture there is no kind of education superior to the scientific, if, indeed, there be any that is equal to it. And the medical student, if he has any capacity for work and study at all, cannot fail of a good scientific education. The medical profession, then, in its learning, in its science, and in its true and noble progress, if not the most honourable of all, is at least a good second to the noblest. It is not with that part of it that any seriously earnest man need quarrel. It is when he comes to practice that the real trials of a man begin.

Now we speak but plain and sober truth when we say that the competition in medical practice at the present time is excessive and ruinous. And this is true of all ranks in the profession. In a very brief article like the present we cannot include all the circumstances bearing upon the case, and therefore, especially as they would convey no intelligent view of the facts to outsiders, we do not give statistics. But what we feel and know is that the vast majority of doctors in full practice have to reckon with a diminishing instead of an increasing income year by year; that old men are steadily elbowed out, and, not having been able to save any money at all in early life, they are often driven to starvation; whilst young men coming in and endeavouring to establish themselves, quite justifiably, in practice against their older rivals, are often compelled to resort to methods which are nothing less than degrading. The following is a generalisation from twenty years' practice in London: Of every six men who take a house and put up a door-plate without buying a practice, five are compelled to leave the house within two years. They leave, with their little capital expended, and with despairing hearts, to renew the struggle, under worse conditions, elsewhere. This is true of London. The writer happens also to have some experience of seaside and other health resorts. If it be possible, the condition of things is decidedly worse there than even in the metropolis. It is heartbreaking to see, as we have seen, in a well-known health resort, middle-aged men, of good character, of sound medical capacity, buying small practices, or introductions to practices, struggling on for two or three years, making no impression upon the public, and finally departing, no one knows where, but often to curse the day when they were tempted to choose medicine as a profession. In the great manufacturing towns, and the factory villages of the north of England, a little more money is, perhaps, to be made. But at what cost? Mostly at the cost of culture, often at the cost of character, and almost invariably at the cost of happiness and health. Then, of agricultural districts—what is to be said? This, that the doctor, like the farmer, the labourer, and, in many cases, even the landlord, is struggling with an annually decreasing income; and has to look forward to an old age for which no provision has been or can be made, and in which, therefore, the same millhorse round must be gone through until the grave gives a last kindly welcome to an always over-worked and a bitterly disappointed man.

Now, we are no pessimists; we believe in work, and in facing the inevitable courageously. This is the lot of the doctor; and to those who are already in physic we say, face it bravely, with a cheerful philosophy, and face it to the end. But to those who are not in we say otherwise. Surely it needs no prophet's warning to make practical young men at least consider facts like these. Perhaps the right thing to say is this: "If you have a keen desire for scientific culture, or an ardent sympathy with your fellow creatures in distress, then by all means think of physic as a possible future calling. But, if your object is merely to earn a living in an honest and respectable way, then give every other honest calling at least a consideration before finally and irretrievably deciding upon a medical career."

